# The Opioid Epidemic Within the COVID-19 Pandemic: Drug Testing in 2020

**DOI:** 10.1089/pop.2020.0230

**Published:** 2021-02-05

**Authors:** Justin K. Niles, Jeffrey Gudin, Jeff Radcliff, Harvey W. Kaufman

**Affiliations:** Quest Diagnostics, Secaucus, New Jersey, USA.

**Keywords:** clinical drug testing, substance use disorder, COVID-19, SARS-CoV-2, opioid, fentanyl

## Abstract

The convergence of the opioid epidemic and the coronavirus disease 2019 (COVID-19) pandemic has created new health care challenges. The authors analyzed changes in clinical drug testing patterns and results at a national clinical laboratory, comparing data obtained before and during the pandemic. Testing for prescription and illicit drugs declined rapidly during the pandemic, with weekly test volumes falling by approximately 70% from the baseline period to the trough (the week beginning March 29) before rising in subsequent weeks. Among individuals tested, positivity increased by 35% for non-prescribed fentanyl and 44% for heroin during the pandemic. Positivity for non-prescribed fentanyl increased significantly among patients positive for other drugs: by 89% for specimens positive for amphetamines; 48% for benzodiazepines; 34% for cocaine; and 39% for opiates (*P* < 0.01 for all comparisons). These findings suggest significant increases in dangerous drug combinations. Positivity for non-prescribed use of many other drugs remained consistent or declined for some drugs, relative to pre-pandemic patterns. Models adjusting for potential confounding variables, including medication-assisted treatment and treatment at a substance use disorder facility indicated that the risk for non-prescribed fentanyl positivity rose by more than 50% during the pandemic. In summary, these findings demonstrate decreased drug testing overall, with increased positivity for high-risk drugs and dangerous drug combinations. The convergence of the drug abuse epidemic and COVID-19 pandemic has led to an increased need for health care and public health resources dedicated to supporting vulnerable patients and addressing the underlying causes of these disturbing trends.

## Introduction

### The drug epidemic

More than 750,000 people have died from a drug overdose in the United States from 1999 to 2018,^1^ with nearly 450,000 deaths involving prescribed or illicit opioids.^2^ The most recent yearly estimates indicate that the overdose epidemic peaked in 2017, with 70,723 reported deaths, followed by a 4.6% decrease in overdose deaths in 2018.^3^ Although progress has been made overall, fentanyl-related deaths threaten to bring deaths from drug overdoses to tragic new heights. The 12-month rolling count of provisional overdose deaths associated with non-methadone synthetic opioids (likely fentanyl) has increased every month since at least January 2015 (5766) through December 2019 (36,509).^4^

### The coronavirus disease 2019 (COVID-19) pandemic

Another public health crisis, the COVID-19 pandemic (national emergency declared on March 13, 2020), has transformed daily life in the United States in innumerable ways. Stay-at-home orders began in various states in March, and by the end of April nearly all states warned their residents to stay home. To help prevent the spread of COVID-19, the Centers for Disease Control and Prevention (CDC) also recommended postponement of medical prevention services that could not be performed virtually.^5^ An unfortunate consequence of these measures was a large decline in clinical drug testing (drug testing to assess compliance with prescribed drugs and/or use of non-prescribed/illicit drugs). Despite the mitigation efforts advocated by medical scientists, businesses, and government agencies, COVID-19 has spread rapidly across the United States, with more than 4.3 million confirmed cases resulting in more than 150,000 deaths as of late July 2020.^6^

### Convergence of an epidemic and pandemic

Nationally, suspected overdose submissions to the Overdose Mapping Application Program (ODMAP) in 2020 rose by 18% in March, 29% in April, and 42% in May, based on a 30-day rolling mean comparison to these months in 2019.^7^ The ODMAP report showed increases of 11.4% for fatal overdoses and 18.6% for nonfatal overdoses during 2020.^8^

According to an estimate by a report from the Well Being Trust and the American Academy of Family Physicians, the economic recession resulting from the COVID-19 pandemic may lead to a large increase in deaths from drug overdose, alcohol abuse, and suicide in coming years.^9^ Depending on the course of the pandemic and response, projections for excess deaths from these causes over the next 10 years range from 27,644 to 154,037, with 75,000 excess deaths being the most likely.^9^ To better understand how the drug epidemic is interacting with the COVID-19 pandemic, the authors investigated changes in the results from clinical drug testing in the United States during the COVID-19 pandemic.

## Methods

This study analyzed urine specimen results from the Quest Diagnostics medMATCH^®^ reporting methodology for clinical drug testing. medMATCH reports indicate whether the prescribed drug(s) specified by the ordering provider, or other drugs, are detected in a specimen.

### Study population

De-identified results from all medMATCH specimens with clinician-provided prescribed drug information performed from January 1, 2019, through May 16, 2020, were selected for potential inclusion. Specimens from patients for whom age was not provided and those younger than 18 years of age were excluded. Specimens with abnormal specimen validity testing results and no drug positivity were excluded as well. Not all specimens were tested for all drug classes, as ordering patterns and perceived medical necessity vary among clinicians ordering testing.

### Definitions

The baseline time period included specimens from January 1, 2019, through March 14, 2020. The COVID-19 pandemic time period included specimens tested March 15 to May 16, 2020. The week starting March 15 was the first full week after a national emergency was declared on March 13. Positivity for non-prescribed drugs was defined as the presence of a positive result for any drugs not listed as prescribed by the ordering clinician, or for recreational/illicit drugs. Noncompliance with prescribed drugs was defined as a negative result for a drug listed as prescribed by the ordering clinician. Drug misuse was defined as either non-prescribed positivity or noncompliance (or both) occurring. When patient state of residence data were not available, the ordering clinician account state was used for analysis. Medication-assisted treatment (MAT) patients were defined using specific *International Statistical Classification of Diseases and Related Health Problems* (ICD-10) F-11 codes. Given the possibility of incomplete ICD-10 code information, non-MAT patients were defined as those both without MAT ICD-10 codes and having negative results for both buprenorphine and methadone. When associated with the diagnostic data, the term patient(s) refers to the subset of people under the care of a physician for the treatment of pain and other conditions whose specimens were tested by Quest Diagnostics.

### Specimen collections and handling

Analysis of all drug testing results included either presumptive immunoassay screening tests, with confirmation of positive results by quantitative definitive mass spectrometry, or tests performed directly by quantitative definitive mass spectrometry. Presumptive immunoassay screening tests were performed using test procedures modified to detect compounds with low cross-reactivity (eg, 7-aminoclonazepam, lorazepam, hydromorphone). Quantitative confirmation analysis was performed to rule out false-positive presumptive screening results. The liquid chromatography-tandem mass spectrometry tests were performed in clinical laboratories and provide definitive quantitative analysis of the drugs and drug metabolites. Mass spectrometry methods were validated using National Institute of Standards and Technology-traceable reference materials.

### Drug metabolites

For analysis of drug positivity, each listed drug included the parent drug(s) and drug metabolite(s). The amphetamines class included both amphetamine and methamphetamine, but not methylenedioxymethamphetamine (which is a separate class). The opiates class included codeine, morphine, hydrocodone, and hydromorphone. The oxycodone class included oxycodone and oxymorphone. A class was counted as positive if any of the parent drugs or metabolites were present.

### Statistical analyses

Statistical significance testing of group positivity proportions was conducted using the chi-square test. Multivariable logistic regression with non-prescribed fentanyl positivity as the dependent variable was performed using a stepwise entry criterion of *P* < 0.05. Specimens missing any values for included variables were not included in the multivariable model analysis. There were 2 US Health and Human Services (HHS) Regions with non-prescribed fentanyl positivity rates higher than the national mean during the baseline time period; these were included as separate binary variables (with all other regions as the reference group) in the multivariable model. Data analyses were performed using SAS Studio 3.6 on SAS 9.4 (SAS Institute Inc., Cary, NC). Quest Diagnostics Health Trends studies are performed on de-identified aggregated data and are deemed exempt by the Western Institutional Review Board (Puyallup, Washington).

## Results

A total of 881,134 specimens were identified that met the criteria for potential inclusion in the study. Specimens were excluded for patients younger than 18 years of age and those without known age (n = 8139). Specimens with abnormal specimen validity testing and no positive drug results (n = 233) also were excluded, leaving a final analytic cohort with 872,762 specimens (99% of potential cohort) from all 50 states and the District of Columbia.

Relative patient demographics of specimens included in the study changed after the baseline period (Table 1). Notable differences included significant decreases in the proportion of specimens from MAT patients and those treated at substance use disorder (SUD) facilities during the COVID-19 pandemic (both *P* < 0.001). There also were significant differences in the proportion of specimens coming from various HHS Regions. Although patient demographics of specimens tested for fentanyl were largely similar to those of all included specimens, specimens tested for fentanyl were more often from MAT patients and SUD facilities (Table 1).

**Table 1.** Demographic Characteristics for All Included Specimens and Fentanyl-tested Specimens, Baseline and During COVID-19

	All included specimens			Fentanyl-tested specimens		
	Before COVID-19	During COVID-19	Sig^*^	Before COVID-19	During COVID-19	Sig^*^
	n (%)	n (%)		n (%)	n (%)	
Total	823,824	48,938		293,253	17,456	
Patient age, years						
18–24.9	13,774 (1.7)	688 (1.4)	<0.001	4,052 (1.4)	194 (1.1)	0.003
25–34.9	83,678 (10.2)	4,315 (8.8)	<0.001	30,836 (10.5)	1,555 (8.9)	<0.001
35–44.9	127,818 (15.5)	7,212 (14.7)	<0.001	45,966 (15.7)	2,630 (15.1)	0.032
45–54.9	168,151 (20.4)	10,326 (21.1)	<0.001	61,869 (21.1)	3,958 (22.7)	<0.001
55–64.9	232,105 (28.2)	14,786 (30.2)	<0.001	85,732 (29.2)	5,435 (31.1)	<0.001
≥65	198,298 (24.1)	11,611 (23.7)	0.083	64,798 (22.1)	3,684 (21.1)	0.002
						
*Sex*						
Male	344,821 (41.9)	20,979 (43.0)	<0.001	123,463 (42.2)	7,702 (44.2)	<0.001
Female	477,926 (58.1)	27,788 (57.0)	<0.001	169,456 (57.9)	9,717 (55.8)	<0.001
						
*MAT status*						
MAT patient	149,202 (18.1)	7,175 (14.7)	<0.001	66,256 (22.6)	3,501 (20.1)	<0.001
Non-MAT patient	635,409 (77.1)	39,464 (80.6)	<0.001	211,361 (72.1)	13,090 (75.0)	<0.001
MAT equivocal	39,213 (4.8)	2,299 (4.7)	0.531	15,636 (5.3)	865 (5.0)	0.031
						
*Facility Type*						
Substance Use Disorder	50,226 (6.1)	2,459 (5.0)	<0.001	22,924 (7.8)	1,387 (8.0)	0.539
General Practitioner	237,356 (28.8)	13,874 (28.4)	0.029	72,868 (24.9)	3,818 (21.9)	<0.001
Pain Management	184,139 (22.4)	15,156 (31.0)	<0.001	88,547 (30.2)	6,378 (36.5)	<0.001
Other	352,103 (42.7)	17,449 (35.7)	<0.001	108,914 (37.1)	5,873 (33.6)	<0.001
						
*Payer Type*						
Medicaid	187,332 (25.4)	11,113 (24.2)	<0.001	75,013 (29.1)	4,708 (29.2)	0.658
Medicare	134,034 (18.2)	9,476 (20.6)	<0.001	45,557 (17.7)	3,318 (20.6)	<0.001
Private Payer	416,323 (56.4)	25,427 (55.3)	<0.001	137,492 (53.3)	8,080 (50.2)	<0.001
						
*Health and Human Services Region*						
1: CT, MA, ME, NH, RI, VT	33,087 (4.0)	1,355 (2.8)	<0.001	20,191 (6.9)	725 (4.2)	<0.001
2: NJ, NY	56,554 (6.9)	1,983 (4.1)	<0.001	21,728 (7.4)	695 (4.0)	<0.001
3: DE, DC, MD, PA, VA, WV	125,946 (15.3)	5,564 (11.4)	<0.001	58,116 (19.8)	2,695 (15.4)	<0.001
4: AL, FL, GA, KY, MS, NC, SC, TN	204,561 (24.8)	14,934 (30.5)	<0.001	63,954 (21.8)	4,350 (24.9)	<0.001
5: IL, IN, MI, MN, OH, WI	114,327 (13.9)	4,789 (9.8)	<0.001	41,617 (14.2)	1,947 (11.2)	<0.001
6: AR, LA, NM, OK, TX	76,489 (9.3)	5,967 (12.2)	<0.001	17,331 (5.9)	1,818 (10.4)	<0.001
7: IA, KS, MO, NE	22,509 (2.7)	2,314 (4.7)	<0.001	8,150 (2.8)	1,182 (6.8)	<0.001
8: CO, MT, ND, SD, UT, WY	1,617 (0.2)	108 (0.2)	0.238	183 (0.1)	8 (0.1)	0.391
9: AZ, CA, HI, NV	165,072 (20.0)	10,693 (21.9)	<0.001	54,460 (18.6)	3,764 (21.6)	<0.001
10: AK, OR, ID, WA	23,502 (2.9)	1,222 (2.5)	<0.001	7,517 (2.6)	271 (1.6)	<0.001

COVID-19, coronavirus disease 2019; MAT, medication-assisted treatment; Sig, significance.

**FIG. 1.**
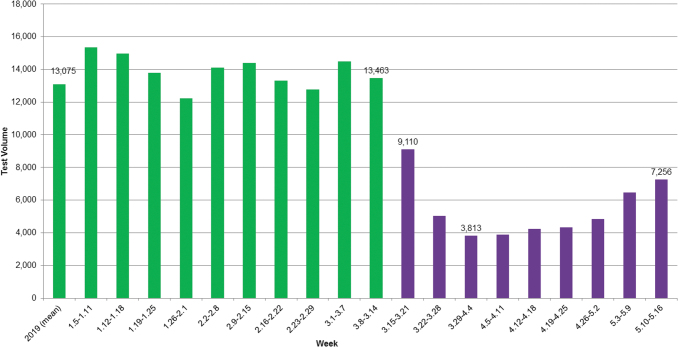
Weekly volume of medMATCH tests with clinician-provided prescription drug(s), 2019 – May 2020. *Green bars* indicate before COVID-19 time period. *Purple bars* indicate during COVID-19 time period. COVID-19, coronavirus disease 2019.

Clinical drug testing declined rapidly during stay-at-home orders, starting the week beginning March 15, 2020 (Figure 1). The weekly clinical drug testing volume fell approximately 70% from the baseline period to the trough (the week starting March 29), and then rose in subsequent weeks. Test volume in the final week of the study had increased more than 90% from the trough but was still 45% below the mean baseline weekly test volume.

**FIG. 2.**
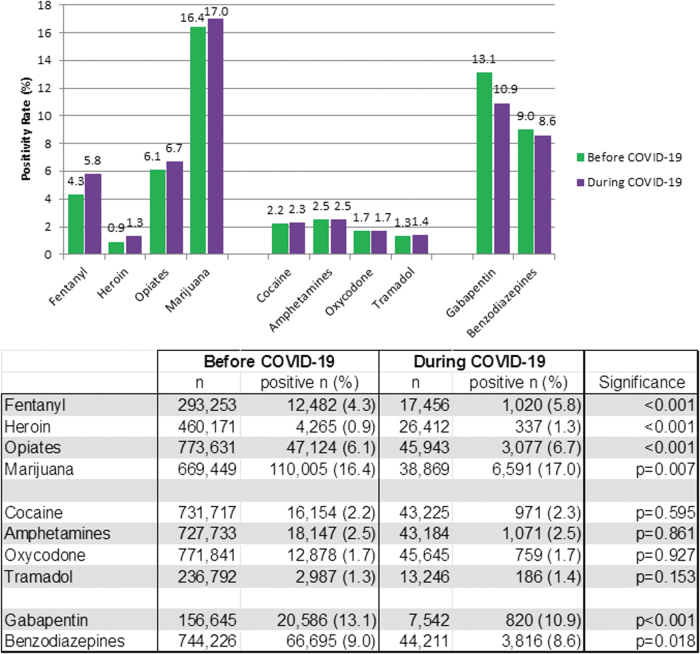
Positivity rates for select non-prescribed drugs, before and during COVID-19 pandemic. Significance analyzed with the chi-square test.

Overall drug misuse was slightly lower during the COVID-19 pandemic (49.4%, 95% C.I. 48.9–49.8%) than the baseline period (49.9%, 95% C.I. 49.8–50.0%, *P* = 0.03), but there were notable exceptions. Positivity for non-prescribed fentanyl increased by 35% (from 4.3% to 5.8%, *P* < 0.01) during the pandemic compared to the baseline period. Significant increases in non-prescribed positivity during COVID-19 also were demonstrated for heroin (44%, *P* < 0.01), opiates (10%, *P* < 0.01), and marijuana (4%, *P* < 0.01) (Figure 2). Offsetting these increases were significant declines in non-prescribed gabapentin (21%, *P* < 0.01) and benzodiazepines (4%, *P* < 0.02) during the pandemic. No significant changes in non-prescribed positivity were found for cocaine, amphetamines, oxycodone, or tramadol.

**FIG. 3.**
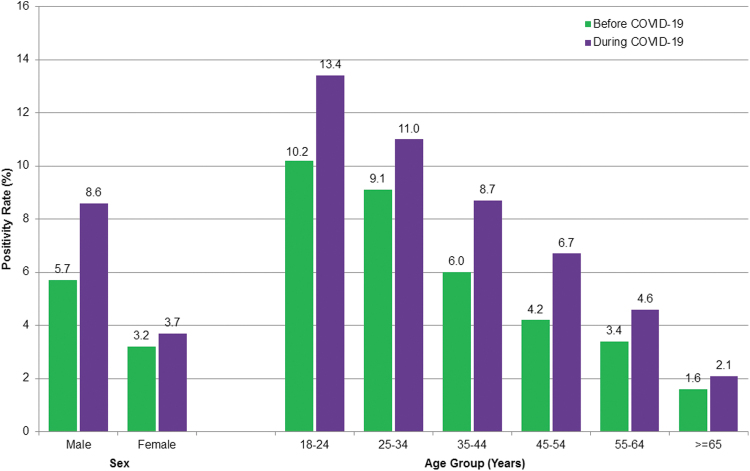
Non-prescribed fentanyl positivity, by age group and sex. Chi-square test indicated significant differences in proportions between time periods for all groups (*P* ≤ 0.02) except the group aged 18–24 years.

Significant increases in non-prescribed fentanyl positivity were not confined to any single patient category: they occurred for both males (51%, *P* < 0.01) and females (16%, *P* < 0.01) and spanned all age groups 25 years and older (*P* = 0.02 or less) (Figure 3). In men younger than 55 years of age, the rate increased by 55%: from 7.8% (95% C.I. 7.6–8.0%) during baseline to 12.1% (95% C.I. 11.0–13.1%) during the pandemic.

**FIG. 4.**
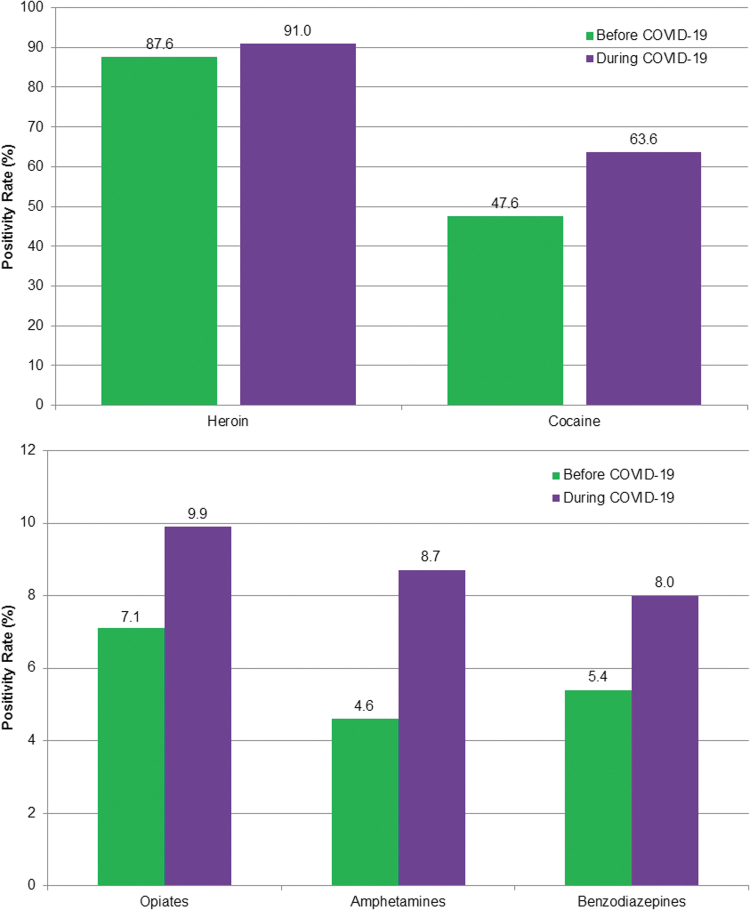
Non-prescribed fentanyl positivity in specimens positive for other drugs. Chi-square test indicated significant differences between time periods for all drug combinations (*P* < 0.01) except heroin.

The combination of non-prescribed fentanyl with other drugs also increased during the pandemic (Figure 4 A and B). Specifically, positivity for non-prescribed fentanyl increased by 89% among patients positive for amphetamines (*P* < 0.01); 48% for benzodiazepines (*P* < 0.01); 34% for cocaine (*P* < 0.01); 39% for opiates (*P* < 0.01); and 4% for heroin. At least 94% of specimens tested for fentanyl also were tested for each of these drugs, both during baseline and during COVID-19, except heroin (80.2%, 95% C.I. 80.1–80.4% during baseline and 75.9%, 95% C.I. 75.3–76.6% during COVID-19).

**FIG. 5.**
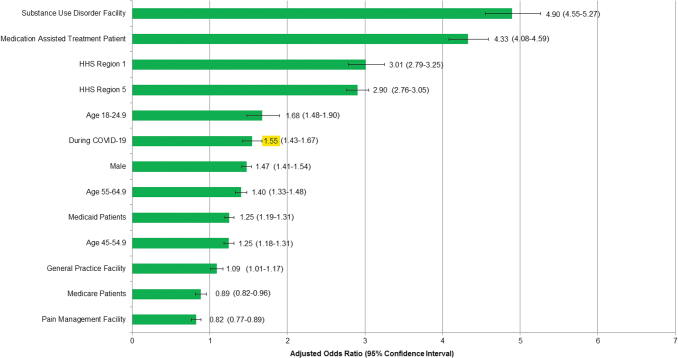
Multivariable logistic regression model: associations of predictive variables with non-prescribed fentanyl positivity. Model included 259,859 of 310,709 fentanyl-tested specimens with no missing values for any factor included in the model. Highlight text shows the Adjusted Odds Ratio of non-prescribed fentanyl during the COVID-19 pandemic. Variables were selected using a stepwise entry criterion of p < 0.05. HHS Region 1 includes specimens from Maine, New Hampshire, Vermont, Massachusetts, Rhode Island, and Connecticut. HHS Region 2 includes specimens from Ohio, Indiana, Illinois, Michigan, Wisconsin, and Minnesota. HHS, Health and Human Services.

In multivariable logistic regression analysis, non-prescribed fentanyl positivity was most strongly associated with MAT patients and treatment at an SUD facility (Figure 5). In contrast, treatment at a pain management facility and being a Medicare recipient were associated with reduced odds of non-prescribed fentanyl positivity. Tests performed during the COVID-19 pandemic remained significantly associated with non-prescribed fentanyl positivity in the model presented (adjusted odds ratio 1.55, 95% C.I. 1.43–1.67) that adjusted for age group, sex, payer type (a surrogate for income in the case of Medicaid), MAT ICD codes, clinician facility type, and select HHS regions.

## Discussion

Study findings indicate that non-prescribed use of the drugs most responsible for the overdose epidemic in recent years (eg, fentanyl) has increased greatly since the start of the pandemic. The increase in non-prescribed fentanyl positivity remained significant in a multivariable model controlling for various independent risk factors. Perhaps even more troubling, the use of fentanyl drug combinations has been increasing as well. These findings are consistent with trends reported previously.^10^ The significant increases in positivity shown for the drugs that cause the most overdose deaths were not demonstrated for most other drug classes. Non-prescribed positivity rates for oxycodone, tramadol, amphetamines, and cocaine did not change significantly during the pandemic.

Numerous factors may be contributing to increased use of certain non-prescribed drugs and dangerous drug combinations during the pandemic. At the onset of the pandemic, to help stop the spread of COVID-19 and prevent further deaths in health care settings, the CDC recommended that health care systems delay elective care.^5^ Although necessary to help mitigate the spread of the pandemic, these orders appear to have led to one of the most dramatic changes brought on by the convergence of the drug epidemic and the COVID-19 pandemic—the temporary discontinuation of testing for drug and alcohol misuse. The result is that many clinicians continue to prescribe controlled substances without drug testing and are therefore “flying blind,” lacking objective evidence to assess their patients for potential drug misuse.

Stress, change, job losses, loneliness, and depression can all trigger prescription medication overuse, illicit drug use, and relapses of drug abuse.^11,12^ Notably, social distancing during the COVID-19 pandemic has isolated vulnerable patients, leaving them to misuse prescription or illicit substances alone. Dr. Nora Volkow, Director of the National Institute on Drug Abuse (NIDA), noted that isolation drives people to initiate drug-taking or relapse.^13^ Moreover, this isolation also makes it less likely that a bystander would be present to call emergency medical services or administer the opioid overdose antidote naloxone. Making matters worse, many SUD treatment centers have been forced to close or scale back significantly during the pandemic shutdowns, leaving less access to these vital services for those in need.^14^ According to a report by the Well Being Trust, “Deaths of despair have been on the rise for the last decade, and in the context of COVID-19, deaths of despair should be seen as the epidemic within the pandemic.”^9^ Given the psychological, social, and financial impacts of the current COVID-19 pandemic, more efforts are needed to ensure that patients are taking medications as prescribed. While the nation focuses its attention on the COVID-19 pandemic, we must not lose sight of the ongoing drug misuse epidemic, which continues to kill more than 67,000 Americans each year.^3^

Positivity for non-prescribed fentanyl showed a marked increase during the pandemic. Fentanyl, a synthetic opioid that is 50 to 100 times more potent than morphine, is a schedule II prescription drug used to treat severe pain. It also is a drug of abuse. However, evidence from drug seizures by the US Drug Enforcement Agency indicates that fentanyl-related harm, overdose, and deaths in the United States are usually the result of illegally made fentanyl, sold through illicit drug markets for its euphoric effect.^15^ Fentanyl is more likely to cause an overdose than is heroin because of its greater potency. In addition, it has a shorter half-life than most other opioids, causing the high to fade more quickly, requiring opioid drug abusers to inject more frequently, and increasing the risk of overdose.

In contrast to fentanyl, non-prescribed gabapentin positivity showed a significant decrease during the pandemic – although non-prescribed positivity remained relatively high, at 10.9%. Gabapentin, an anticonvulsant approved for the treatment of neuropathic pain, is often prescribed as an alternative to opioids for managing chronic pain. When taken alone and as prescribed, there is little potential for misuse or addiction. However, when taken with other medications, such as muscle relaxants, opioids, or anxiety medications, it can produce a feeling of euphoria, sedation, or high. A possible contributing factor for the decline in non-prescribed use of gabapentin during the pandemic is declining physician visits – with fewer prescriptions, these drugs may become less available for misuse through diversion.

Drug combinations remain a significant source of mortality from illicit drug use. According to the CDC, benzodiazepines, cocaine, or methamphetamines were present in 63% of recent opioid deaths.^16^ Fentanyl is often mixed with other illicit drugs—with or without the user's knowledge. These combinations are especially dangerous because of the high potency of fentanyl, meaning that even a small quantity has a powerful effect on depressing respiration. Quest Diagnostics previously reported high rates of non-prescribed fentanyl found in individuals using cocaine or heroin.^10^ The overwhelming majority of specimens positive for heroin also were positive for non-prescribed fentanyl, and this has increased even further during the pandemic. Non-prescribed fentanyl was found in nearly half (48%) of specimens positive for cocaine before stay-at-home orders were implemented, and increased to nearly two thirds (64%) since. Although occurring at lower overall rates, positivity for non-prescribed fentanyl has increased dramatically in specimens positive for opiates, amphetamines, or benzodiazepines as well. In the case of amphetamines, there was a nearly 90% relative increase during the COVID-19 pandemic. The potential impact of these combinations on both overdose hospital admissions and overdose deaths is important. At a time when hospital beds in COVID-19 “hot zones” can be in short supply, concurrent increases in the need for hospital admissions related to drug overdoses may have grim consequences.

Certain demographic characteristics of the population tested changed during the COVID-19 pandemic. The change in sex distribution was minor and likely of little clinical significance. However, changes in other features, including a decline in specimens from MAT patients and a marked increase in the proportion of specimens from pain management facilities, were noteworthy. There also were significant differences in the proportion of specimens from various HHS regions, likely reflecting geographic differences in the timing of stay-at-home orders and the differing impact of COVID-19. The authors expected that disproportionate demographic and patient risk changes would have had a large impact on study results. However, in the multivariable model that adjusted for age group, sex, payer type (a surrogate for income in the case of Medicaid), MAT ICD codes, clinician facility type, and select HHS regions, it was found that simply comparing the baseline and during COVID-19 rates may not fully estimate the true impact of COVID-19 on non-prescribed fentanyl positivity. The adjusted model indicated a 55% increase in risk of non-prescribed fentanyl positivity associated with specimens processed during the COVID-19 pandemic, higher than the 35% increase found by comparing baseline to during COVID-19. The higher risk in the adjusted model likely is driven by the significant decrease in the proportion of specimens from MAT patients and specimens from US HHS regions 1 (New England) and 5 (Ohio, Indiana, Illinois, Michigan, Wisconsin, and Minnesota), which are 3 of the strongest factors associated with non-prescribed fentanyl positivity during the COVID-19 pandemic. This would, in theory, have driven the positivity rate lower. A key question raised by present study findings is whether the increased positivity rates for fentanyl and some other drugs are temporary, lasting just during the early stage of the COVID-19 pandemic, or if they signify a longer lasting shift in drug misuse behavior.

Strengths of this study include its use of validated testing by mass spectrometry, the most sensitive and specific drug testing method, as well as its size and geographic scope. Limitations of this study include geographic disparities and the inability to validate or contextualize test results with clinical history, including overdose and hospitalization. To provide the most stable baseline data for comparison, the baseline period was designed to be considerably longer than the COVID-19 study period. Although this design led to differences in the seasons included in each period, the authors have not observed seasonal variations in drug misuse in the 8 years that Quest Diagnostics has been reporting clinical drug test results (data not shown). In a small minority of specimens, patient-level variables (eg, hydration state, drug metabolism) and methodology limitations can contribute to a failure to detect drugs. Patients were referred for testing by their health care providers to evaluate drug compliance or misuse and the selection of such patients during the pandemic is unknown. In addition, some health care providers may have neglected to indicate all prescribed drugs a patient was taking when submitting the test request, which would affect the medMATCH analysis. Some demographic characteristics of the population tested shifted after the stay-at-home period, but the authors conducted multivariable analyses adjusting for all the relevant factors possible to ensure that the main outcomes were not simply a function of these changes. These results are reflective of the population being monitored for drug misuse and adherence but are not necessarily reflective of the general population.

## Conclusions

Present study findings indicate that clinical drug testing decreased during the COVID-19 pandemic, while non-prescribed positivity for fentanyl and many fentanyl drug combinations has increased significantly. Now, perhaps more than ever, given the increased stressors associated with the pandemic, we must maintain extra vigilance and not lose ground in our continued efforts to combat our nation's drug abuse crisis. The authors recommend a renewed focus in providing education and support to treating clinicians on the front lines, including reinforcing the importance of drug testing, the only objective measure of what drugs or substances patients are taking.
